# Hepatitis A serostatus and vaccination outcomes after on-campus education and point-of-care antibody testing

**DOI:** 10.3389/fpubh.2026.1740378

**Published:** 2026-03-11

**Authors:** Jun Hwi Cho, Jin Kim, Ran Lee, So Hyun Bae, A Ram Park, Jang Gwon Yoon, Ji In Seo, Hye Rin Na, Kyung-Hwa Park, So Yeon Ryu, Seong Eun Kim

**Affiliations:** 1 Gwangju Center for Infectious Diseases Control and Prevention, Gwangju, Republic of Korea; 2 Department of Public Health, Graduate School, Chonnam National University, Gwangju, Republic of Korea; 3 Department of Infectious Diseases, Chonnam National University Medical School, Gwangju, Republic of Korea; 4 Department of Preventive Medicine, Chosun University Medical School, Gwangju, Republic of Korea

**Keywords:** adult immunization, hepatitis A, point-of-care testing, public health intervention, vaccination uptake

## Abstract

**Background:**

Susceptibility to hepatitis A virus (HAV) remains high among young adults in Korea, reflecting birth-cohort effects and vaccination history. Because foodborne transmission has contributed to outbreaks, students in food and nutrition or culinary arts represent a relevant target group as future food handlers. We aimed to estimate anti-HAV immunoglobulin G (IgG) seroprevalence, describe pre- to post-intervention changes in knowledge, perceptions and vaccination intention and report two-dose vaccination completion and its correlates.

**Methods:**

From April 2023 to December 2024, we conducted a campus-based hepatitis A prevention program at four universities. Students completed a baseline questionnaire before the session and a post-intervention questionnaire immediately after the education session and same-visit POCT result feedback. Students who were IgG-negative and had no verified prior two-dose vaccination in the national Immunization Registry Information System (IRIS) were referred to public health centers for no-cost vaccination. Two-dose completion was defined as IRIS documentation of a second HAV vaccine dose by the study end date. We modeled anti-HAV IgG positivity using mixed-effects logistic regression with an institution-level random intercept, and series completion (and correlates) using modified Poisson regression with institution-clustered robust standard errors.

**Results:**

Of 692 participants who underwent anti-HAV IgG POCT, 188 (27.2%) were IgG-positive and 504 (72.8%) IgG-negative. Among IgG-negative participants, 37 had documented prior two-dose vaccination and were not vaccine-eligible. The remaining 467 were vaccine-eligible (0 dose *n* = 435, 1 dose *n* = 32). Overall, 354/467 (75.8%) completed the two-dose series. Baseline intention was strongly associated with series completion (aRR 1.55; 95% CI, 1.39–1.72). Among those initially no/undecided (*n* = 114), endorsing vaccine distrust was associated with higher completion (aRR 1.46; 95% CI, 1.09–1.98).

**Conclusion:**

In a cohort of future food handlers with a susceptibility gap, a campus-based program integrating standardized education with same-visit anti-HAV IgG testing and vaccination record verification achieved high two-dose completion among those without evidence of protection. These findings inform targeted young-adult hepatitis A prevention strategies by using on-site triage to focus counseling and referral on susceptible individuals and reduce reliance on off-site testing and return visits. Comparative studies are needed to assess the incremental value and cost-effectiveness of on-site testing and serostatus feedback.

## Introduction

Hepatitis A virus (HAV) remains common in low- and middle-income countries, where early-life exposure yields lifelong immunity. When sanitation improves and early-life HAV exposure declines, population immunity shifts to older ages, leaving more adolescents and adults susceptible and increasing the potential for outbreak in higher-income settings (1, 2). Many countries therefore recommend hepatitis A vaccination to protect unimmunized cohorts (3).

In Korea, routine childhood vaccination was introduced for children born after 2012 (4), yet immunity gaps persist in young adults. National data show substantially higher seropositivity in adolescents than in people in their twenties, indicating suboptimal protection among those entering the workforce (5, 6). Reflecting these susceptibility gaps in young adults, Korea experienced major hepatitis A outbreaks in 2009 and again in 2019, with 15,231 and 17,598 reported cases, respectively. The 2019 resurgence was linked to foodborne transmission from contaminated salted clams (7, 8). Given the ongoing susceptibility gap in young adults and the documented potential for foodborne transmission, university students majoring in food and nutrition or culinary arts represent a preventive target group as future food handlers.

Beyond their relevance as future food handlers, university students are a key population for adult hepatitis A prevention because they belong to young adult cohorts with well-documented immunity gaps and suboptimal uptake of recommended adult vaccines. Vaccine hesitancy in young adults, including college-aged populations, has been linked to confidence-related concerns and perceived low personal risk, which can undermine vaccine acceptance (9, 10). In this context, university-based programs offer a practical platform to deliver standardized education, provide individualized risk or serostatus feedback, and facilitate access to vaccination before students enter food service or other public-facing occupations.

We implemented a program that delivered standardized education with on-site anti-HAV immunoglobulin G (IgG) point-of-care testing (POCT) to inform individualized vaccination decisions. In this study, we aimed to estimate anti-HAV IgG seroprevalence, describe pre- to post-intervention changes in knowledge, perceptions, and vaccination intention, and report two-dose vaccination completion and its correlates.

## Methods

### Study design and participants

This study was conducted as part of a hepatitis A prevention program implemented by the Gwangju Center for Infectious Diseases Control and Prevention (GCIDC). We invited students from food-related departments (Food and Nutrition and Culinary Arts) at four universities in Gwangju, South Korea (Apr 2023-Dec 2024) to participate in the program, and enrolled 698 students who provided consent to receive hepatitis A education and undergo on-site anti-HAV IgG POCT. Of the 698 enrolled participants, 4 did not complete the baseline survey and 2 did not have POCT results and were excluded. This resulted in an analytic sample of 692 participants who completed the baseline survey and underwent on-site anti-HAV IgG POCT. These 692 participants were included in the main analyses.

### Intervention: standardized education and on-site POCT

The intervention comprised two components delivered during the same campus visit: (i) a standardized, staff-led education session on hepatitis A (epidemiology, transmission, clinical features, vaccine effectiveness, and prevention behaviors) and (ii) on-site anti-HAV IgG POCT. POCT results were provided to participants during the visit. An English summary of the education content is provided in Supplementary material S1.

### Questionnaires and measures (baseline and post-intervention)

We administered two time-point questionnaires during the campus visit. A baseline questionnaire completed before the education session and POCT, and a post-intervention questionnaire completed immediately after completion of both components and receipt of POCT results. The questionnaires assessed hepatitis A related knowledge, perceptions/attitudes regarding hepatitis A and vaccination, preventive behaviors, and vaccination intention. Vaccination intention and reasons for hesitancy were assessed at baseline only.

Knowledge was assessed using 13 dichotomous items and summarized as a total score ranging from 0 to 13. Perceptions were assessed using four numeric rating scale items (1–10) and analyzed on their original scales. Preventive behaviors were assessed at baseline using 10 numeric rating scale items (1–10) and summarized as the mean score. Vaccination intention was assessed at baseline and coded for analysis as yes versus no/undecided. Among participants reporting no/undecided, reasons for hesitancy were collected at baseline (multiple responses allowed) and were grouped into three categories (“vaccine distrust,” “lack of knowledge,” and “other reasons”) for analysis. Reasons were originally captured using six prespecified response options (distrust, perceived low risk, medical concern, lack of knowledge, cost, and other), and were collapsed into three analytic categories because several options were infrequently endorsed to improve interpretability and model stability.

For domains assessed at both time points (knowledge and perceptions), item wording and response options were identical to enable baseline-to-post-intervention comparisons. Full item wording and response options are provided in Supplementary material S2 (baseline) and S3 (post-intervention).

The questionnaires were developed for this program by the GCIDC and the study team, guided by health behavior theory constructs and a vaccine hesitancy framework, and informed by prior literature on vaccine hesitancy and related survey domains (11–13). The questionnaires were reviewed by an infectious disease physician and a preventive medicine specialist for clarity and content relevance. No formal pretesting was conducted. Because both components were delivered during a single visit, pre–post changes reflect the overall program experience and cannot be attributed to education or POCT alone.

### Point-of-care anti-HAV IgG testing

Hepatitis A antibody status was assessed on-site using the BIOCREDIT HAV IgG rapid diagnostic kit (RapiGEN Inc., Suwon-si, Republic of Korea; Cat No. A10RHA25), which has a sensitivity of 99.6% and specificity of 99.8% per manufacturer’s instructions. A 20 μL whole-blood sample was applied and results read at 15 min per the manufacturer’s instruction. Results were classified as IgG-positive (two visible lines) or IgG-negative (control line only).

### Vaccination record verification and protocol

After the intervention, vaccination histories were verified in the national Immunization Registry Information System (IRIS) operated by the Korea Disease Control and Prevention Agency, to determine prior hepatitis A doses. Vaccine eligibility was defined as anti-HAV IgG-negative and without confirmed completion of the two-dose hepatitis A series in IRIS (i.e., 0 or 1 documented prior dose). When anti-HAV IgG results and vaccination records were discordant (e.g., IgG-negative despite IRIS-documented completion of the two-dose series), individuals were not offered revaccination through the program, as IgG-negativity may reflect waning antibody levels after vaccination. Beginning 5 weeks after the intervention, designated public health centers operated a four-week first dose campaign. Participants with a single documented prior dose were offered dose 2 during this period. The second-dose campaign was conducted ≥6 months after dose 1 and ran for 4 weeks. The primary endpoint, series completion, was defined as documented receipt of dose 2 by the study end date. Verification was conducted through the designated public health centers. All vaccination costs were covered by the GCIDC hepatitis A prevention program.

### Statistical analysis

Continuous variables were summarized as means with standard deviations and categorical variables as counts with percentages. Group comparisons used independent two-sample t-tests and chi-square tests (Table 1). Anti-HAV IgG positivity was modeled using mixed-effects logistic regression with a random intercept for institution to account for clustering (Table 2). For Table 2, adjusted models included age, sex, current smoking, alcohol use, self-reported prior HAV disease/contact history, self-reported hepatitis A vaccination, and military-cook experience entered simultaneously. Two-dose series completion analyses were restricted to vaccine-eligible participants (as defined above). Completion was modeled using modified Poisson regression with a log link to estimate risk ratios, with institution-clustered robust standard errors (Tables 3, 4). We used mixed-effects logistic regression for seropositivity as a standard approach for binary outcomes while accounting for institutional clustering, whereas we used modified Poisson regression for series completion to directly estimate risk ratios because completion was common and odds ratios could overstate relative differences. For Tables 3, 4, models were adjusted for age and sex. The same approach was applied to analyses restricted to participants who were hesitant at baseline. Post-intervention outcomes (knowledge and perceptions) were compared using analysis of covariance with a random intercept for institution, adjusting for the corresponding baseline value, age, and sex (Table 5). Analyses used complete-case data, and no adjustment was made for multiple comparisons. Analyses were performed using SAS 9.4 (SAS/STAT 15.3, SAS Institute Inc., Cary, NC, USA). All tests were two-sided and *p* < 0.05 was considered statistically significant.

**Table 1:** Baseline characteristics by anti-HAV IgG POCT result.

Characteristic	Total (*n* = 692)	HAV IgG POCT-positive (*n* = 188)	HAV IgG POCT-negative (*n* = 504)	*p*-value
Age, years (mean ± SD)	20.93 ± 2.37	21.05 ± 2.12	20.76 ± 2.43	0.03
Male, n (%)	205 (29.6)	89 (47.3)	116 (23.0)	<0.001
Current smoking, *n* (%)	125 (18.1)	47 (25.0)	78 (15.5)	0.004
Alcohol use ≥1 time/month, *n* (%)	534 (77.2)	144 (76.6)	390 (77.4)	0.83
Self-reported HAV disease/contact history, *n* (%)	13 (1.9)	6 (3.2)	7 (1.4)	0.12
Self-reported HAV vaccination history, *n* (%)	49 (7.1)	23 (12.2)	26 (5.2)	0.001
History of military service, *n* (%)	128 (18.5)	73 (38.8)	55 (10.9)	<0.001
Military-cook experience, *n* (%)	53 (7.7)	42 (22.3)	11 (2.2)	<0.001

HAV, hepatitis A virus; IgG, immunoglobulin G; POCT, point-of-care test.

*p*-values from *t*-test (continuous) and chi-square (categorical). Age is shown as mean ± SD. All other variables were coded as binary and counts shown are for the ‘yes’ category.

**Table 2:** Factors associated with hepatitis A IgG POCT positivity among study participants.

Covariate	Adjusted odds ratio (95% CI)	*p*-value
Age (per 1-year increase)	0.93 (0.85–1.02)	0.11
Male	1.86 (1.20–2.88)	0.006
Current smoking	1.02 (0.62–1.68)	0.95
Alcohol use ≥1 time/month	0.79 (0.51–1.22)	0.29
History of HAV disease or contact	2.59 (0.81–8.30)	0.11
Self-reported HAV vaccination history	1.42 (0.70–2.84)	0.33
Military-cook experience	9.56 (4.37–20.88)	<0.001

HAV, hepatitis A virus; IgG, immunoglobulin G; POCT, point-of-care test.

Values are adjusted odds ratios with 95% CIs from mixed-effects logistic regression with a random intercept for institution. Age modeled as continuous; other covariates were binary indicators. Military service was excluded due to collinearity with military-cook experience. *p*-values are shown for each estimate.

**Table 3:** Baseline intention and series completion among 467 vaccine-eligible participants.

Baseline intention	Completed, *n* (%)	Adjusted relative risk (95% CI, *p*-value)	Risk difference, percentage points (95% CI, *p*-value)
Yes (*n* = 353)	292 (82.7)	1.55 (1.39–1.72; *p* < 0.001)	26.02 (20.2–31.9; *p* < 0.001)
No/Undecided (*n* = 114)	62 (54.4)	ref	ref

Vaccine-eligible was defined as anti-HAV IgG-negative on POCT and without verified two-dose completion in the national Immunization Registry Information System (IRIS) at baseline. Adjusted relative risks were estimated using modified Poisson regression (log link) controlling for age and sex with institution-clustered robust standard errors. Row percentages are based on row totals. ‘Ref’ indicates the reference category.

**Table 4:** Baseline intention reasons for hesitancy and series completion among the 114 participants whose baseline intention was no or undecided.

Reason	Endorsed the reason		Not endorsed the reason		Adjusted relative risk (95% CI)	*p*-value
	Total, *N*	Completed, *n* (%)	Total, *N*	Completed, *n* (%)		
Vaccine distrust	18	14 (77.8)	96	48 (50.0)	1.46 (1.09–1.98)	0.01
Lack of knowledge of hepatitis A	53	32 (60.4)	61	30 (49.2)	1.16 (0.88–1.51)	0.29
Others	68	31 (45.6)	46	31 (67.4)	0.74 (0.49–1.11)	0.14

Restricted to participants whose baseline intention was no or undecided. Multiple responses permitted. Adjusted Relative Risk were estimated using modified Poisson regression (log link) adjusted for age and sex with institution-clustered robust standard errors. Completion denotes IRIS-verified receipt of dose 2. ‘Others’ include medical concern (adverse reaction), cost, and perceived low risk.

**Table 5:** Post-intervention perceptions and knowledge among 467 vaccine-eligible participants.

Outcome	Completed (*n* = 354), adjusted mean	Not completed (*n* = 113), adjusted mean	Adjusted mean difference (95% CI)	*p*-value
Disease knowledge	7.56	7.09	0.47 (0.08–0.86)	0.02
Vaccine knowledge	7.65	7.14	0.52 (0.12–0.91)	0.01
Education need	8.22	7.91	0.31 (−0.06–0.68)	0.10
Vaccine trust	8.02	7.52	0.51 (0.18–0.84)	0.003
Objective knowledge score	11.60	11.38	0.22 (−0.05–0.48)	0.11

Post-intervention outcomes were measured immediately after the intervention and are stratified by final vaccination completion status (completed vs not completed). Adjusted marginal means were estimated with analysis of covariance (ANCOVA) models that included a random intercept for institution and adjusted for the corresponding baseline value, age, and sex. Kenward-Roger degrees of freedom were used. The self-reported outcomes were scored from 1 to 10 and the objective knowledge score from 0 to 13. The adjusted mean difference compares the Completed group with the Not completed group (positive values indicate higher post-intervention scores among completers).

## Results

### Participant flow and vaccine eligibility

**Figure 1:**
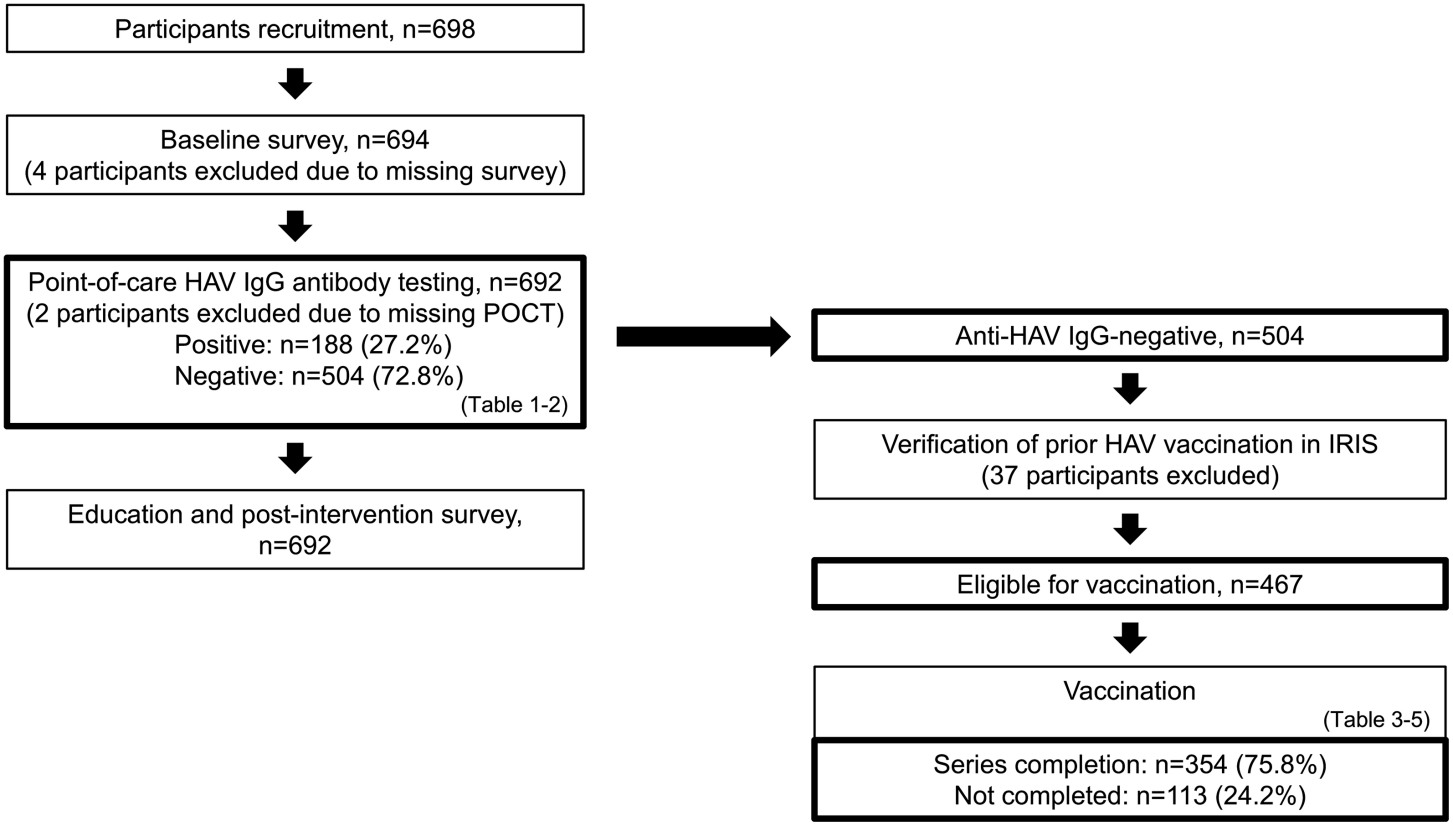
Participant flow from recruitment to verified vaccination outcomes. Flow diagram showing sample counts at each stage. Recruited (*n* = 698). Baseline survey (*n* = 694). Education with on-site anti-HAV POCT (*n* = 692). Among those tested, IgG-positive were 188 and IgG-negative were 504. After IRIS verification, 37 individuals with a previously completed two-dose series were excluded, leaving 467 vaccine-eligible participants (0 prior dose *n* = 435, 1 prior dose *n* = 32). Among the eligible group, 354 completed the series and 113 did not. HAV, hepatitis A virus; IgG, immunoglobulin G; POCT, point-of-care test; IRIS, Immunization Registry Information System.

Among 698 students enrolled, 692 completed the baseline survey, received the standardized education, and underwent on-site anti-HAV IgG POCT and were included in the analyses (Figure 1). Of these 692 participants, 188 (27.2%) were IgG-positive and 504 (72.8%) were IgG-negative. Among IgG-negative participants, IRIS verification identified 37 IgG-negative individuals with documented completion of a prior two-dose hepatitis A vaccine series. These individuals were not included in analyses of series completion. The remaining 467 participants met vaccine-eligibility criteria (435 with no prior hepatitis A vaccination and 32 with a single prior dose). Among vaccine-eligible participants, 354/467 (75.8%) completed the hepatitis A vaccine series and 113/467 (24.2%) did not (Figure 1).

### Seroprevalence and factors associated with anti-HAV IgG positivity

Baseline characteristics by POCT result are shown in Table 1. Participants with positive IgG were slightly older (mean 21.05 vs. 20.76 years; *p* = 0.03), more often male (47.3% vs. 23.0%; *p* < 0.001), more frequently current smokers (25.0% vs. 15.5%; *p* = 0.004), and more likely to report military-cook experience (22.3% vs. 2.2%; *p* < 0.001). Self-reported hepatitis A vaccination history was also more common in the IgG-positive group (12.2% vs. 5.2%; *p* = 0.001). Other variables were not significantly different.

Adjusted associations with IgG positivity are shown in Table 2. Adjusted models included the prespecified covariates listed in the Statistical Analysis section, entered simultaneously. Military-cook experience showed the strongest association with IgG positivity (adjusted OR 9.56; 95% CI, 4.37–20.88; *p* < 0.001), and male sex remained independently associated (adjusted OR 1.86; 95% CI, 1.20–2.88; *p* = 0.006). Age, smoking, alcohol use, self-reported prior disease/contact, and self-reported vaccination were not independently associated with IgG positivity in the adjusted model.

### Vaccination uptake among vaccine-eligible participants

Among the 467 vaccine-eligible participants, the overall series completion rate was 75.8% (354/467). Of the 354 completers, 322 had no documented prior vaccination and completed both doses during the study, whereas 32 had a single documented prior dose and completed the remaining second dose.

Associations with series completion are shown in Table 3. Adjusted estimates were obtained from models including age and sex as covariates. Participants who intended to vaccinate at baseline were more likely to complete the series than those who were no or undecided (82.7% vs. 54.4%); this corresponded to an adjusted relative risk (aRR) of 1.55 (95% CI, 1.39–1.72; *p* < 0.001) and a risk difference of 26.02 percentage points (95% CI, 20.2–31.9; *p* < 0.001, Table 3).

Among the subset with no or undecided intention at baseline (*n* = 114), endorsement of vaccine distrust as a reason was associated with higher completion (77.8% vs. 50.0%; aRR 1.46; 95% CI, 1.09–1.98; *p* = 0.01), while endorsing lack of hepatitis A knowledge (60.4% vs. 49.2%; aRR 1.16; 95% CI, 0.88–1.51; *p* = 0.29) or other concerns (45.6% vs. 67.4%; aRR 0.74; 95% CI, 0.49–1.11; *p* = 0.14) was not significantly associated. These subgroup comparisons were exploratory (Table 4).

### Post-intervention perceptions and knowledge by completion status

Adjusted post-intervention means favored the Completed group for several outcomes (Table 5). Between-group adjusted mean differences were 0.51 (95% CI, 0.18–0.84; *p* = 0.003) for vaccine trust, 0.52 (95% CI, 0.12–0.91; *p* = 0.01) for vaccine knowledge, and 0.47 (95% CI, 0.08–0.86; *p* = 0.02) for disease knowledge. Differences for education need (0.31; 95% CI, −0.06 to 0.68; *p* = 0.10) and the objective knowledge score (0.22 on a 0–13 scale; 95% CI, −0.05–0.48; *p* = 0.11) did not meet statistical significance.

## Discussion

We evaluated a campus-based hepatitis A prevention program that integrated standardized education, on-site anti-HAV immunoglobulin G (IgG) point-of-care testing (POCT) and facilitated referral to no-cost vaccination for students in food-related majors. In this cohort, approximately one-quarter of participants were anti-HAV IgG positive, and roughly three-quarters of vaccine-eligible students completed the two-dose series. Completion was higher among students who reported an intention to vaccinate at baseline, which was assessed before the education session and POCT.

All participants were born before the birth cohorts covered by Korea’s National Immunization Program introduced in 2012. Routine childhood HAV vaccination therefore did not apply to this cohort (4). The seropositivity observed in our participants (27.2%) was substantially higher than prior reports for similar ages in Korea. A study conducted between 2010 and 2014 reported 12.7% seropositivity among individuals aged 20 to 24 years (5). This higher seropositivity may reflect vaccination opportunities outside routine pediatric programs, particularly those related to military service, as well as cohort characteristics. Since 2012, the Republic of Korea Armed Forces have administered one dose of hepatitis A vaccine to all new recruits and a two-dose schedule to personnel in food-service roles, including military cooks, and to medical staff (14). National surveillance has also reported sex differences in hepatitis A incidence in Korea (15). Taken together, these observations provide context for the higher seropositivity observed in this cohort. Because vaccination opportunities in Korea may vary by sex and military service history, differences in participant characteristics could have contributed to this pattern. However, these factors were not the primary focus of the present study and should be interpreted descriptively.

Among vaccine-eligible students, 75.8% (354/467) completed the two-dose hepatitis A vaccine series. Our program delivered education, immediate serostatus information via POCT, and a structured pathway to free vaccination as an integrated package. Because this was a single-arm evaluation, we could not determine the independent contribution of each component to series completion. Although direct comparisons across studies are limited by differences in design and delivery contexts, the vaccine completion level appears comparable to some targeted adult hepatitis vaccination programs that combine education with facilitation (16, 17).

Vaccine hesitancy is widely recognized as an important determinant of delayed or incomplete vaccination in adult populations and confidence and knowledge-related concerns are commonly cited drivers (9). In college-aged and young adult populations, hesitancy may contribute to missed vaccination opportunities, particularly during periods of training that precede entry into food service and other public-facing occupations. Correspondingly, education and communication strategies that address these domains have been evaluated as approaches to support vaccine uptake in some settings (18). In college-aged populations, intervention discussions also emphasize reducing practical barriers through convenient access and offering individualized information at the point of contact to support decision-making (10, 18). In this context, the same visit serostatus information provided by on-site POCT may reduce uncertainty about one’s immune status and could help some individuals resolve indecision at the point of contact. However, our design does not allow isolation of the effect of serostatus feedback on hesitancy or uptake. Further comparative research is needed to evaluate whether immediate serostatus feedback provides incremental benefit beyond education and facilitated access alone.

From a policy perspective, Korea’s routine childhood hepatitis A immunization program, introduced for birth cohorts after 2012, does not directly address the large susceptible pool among those born earlier who continue to enter food-related training and the workforce. In a country that has experienced periodic hepatitis A outbreaks, these birth-cohort gaps support consideration of complementary strategies for young adults, such as targeted catch-up vaccination or screening-and-vaccinate approaches in settings with higher transmission relevance, including future food handlers.

In settings where immunity and vaccination history are mixed and documentation may be incomplete, on-site anti-HAV IgG POCT can serve an operational role at first contact (19). By separating immune from susceptible students during the campus visit and pairing results with registry verification, programs can focus counseling and referral on those without evidence of protection. This approach may help avoid vaccinating individuals who already have evidence of protection compared with a blanket recommendation approach (3, 19). The program also illustrates a pragmatic resource-allocation strategy. Same-visit triage can concentrate counseling, referral efforts, and vaccine supply on susceptible individuals rather than delivering resources uniformly. This may reduce unnecessary vaccination and decrease the operational burden associated with off-site laboratory testing and return visits for results (20).

Attitudinal findings provide additional context for future program development. Students who intended to vaccinate at baseline were more likely to complete the series. Among those who were undecided or not intending at baseline, completion varied by stated reasons for hesitancy. Endorsement of vaccine distrust before the intervention was associated with higher completion than endorsement of lack of knowledge or other reasons. Post-intervention ratings of vaccine trust and vaccine knowledge were higher among eventual completers. Communication strategies that address confidence and knowledge have been evaluated in prior vaccine-uptake interventions (9, 18). These subgroup findings should be interpreted cautiously given the limited sample size and the exploratory nature of these analyses.

Limitations should be considered. Because this was a single-arm evaluation, the observed completion and associated factors should be interpreted descriptively. The study design does not allow estimation of the independent contributions of education, POCT and serostatus feedback, referral procedures, or removal of financial barriers. The study was conducted in a single city and participation was voluntary, which may limit generalizability to other regions or student populations. Program performance may differ in settings with different baseline HAV immunity, vaccination opportunities such as military service, access to public health centers, or availability of no-cost vaccination. Some baseline characteristics were self-reported. Post-intervention perceptions were assessed immediately after the campus visit, whereas series completion occurred over subsequent months. Observed differences by completion status therefore reflect associations and may be influenced by unmeasured factors. Future comparative evaluations, such as otherwise identical education and referral programs delivered with versus without on-site anti-HAV IgG POCT, could help estimate the incremental value of serostatus feedback while holding other supports constant.

In summary, in a cohort of future food handlers, we observed high two-dose completion among vaccine-eligible students in a campus-based program that integrated standardized education, on-site anti-HAV IgG testing, and structured referral to free vaccination. Completion was higher among those with baseline intention to vaccinate. Subgroup patterns in reasons for hesitancy and post-intervention ratings also highlight communication targets that merit further evaluation in comparative and implementation studies. These findings may inform targeted hepatitis A prevention approaches for young adult cohorts with documented susceptibility gaps, including students preparing for food-handling occupations. A campus-based one-stop model that combines brief education, on-site anti-HAV IgG testing with immunization record verification, and facilitated referral to no-cost vaccination may help focus efforts on those without evidence of protection, although comparative evaluations are needed to assess incremental value and cost-effectiveness.

## Data Availability

The datasets presented in this study can be found in online repositories. The names of the repository/repositories and accession number(s) can be found at: https://doi.org/10.7910/DVN/0UEIYP.
